# Trueness of Intraoral Scanners for Edentulous Mandibular Arches With and Without Landmarks

**DOI:** 10.4317/jced.63287

**Published:** 2025-10-17

**Authors:** Filip Rebelo Dessborn, Michael Braian

**Affiliations:** 1 DDS. Swecadd and Baltzar Tandvård, Malmö, Sweden. Orcid-ID. 0009-0007-7640-38842; 2 DDS, PhD. Swecadd and Baltzar Tandvård, Malmö, Sweden

## Abstract

**Background:**

The purpose of this in vitro study was to evaluate the trueness of three intraoral scanners (IOS) and to assess the impact of geometrical landmarks on the digitization of edentulous mandibular complete-arch casts using a standardized scanning protocol.

**Material and Methods:**

A 3D-printed edentulous mandibular cast with five cylindrical landmarks was scanned using three IOS systems (Medit i700, Primescan AC and Trios 5) under two conditions: with and without geometrical landmarks (GL/NG). Each scanner performed 15 scans per condition. A coordinate measuring machine (CMM) provided reference values for cross-arch and inter-cylindrical distances. STL files were analyzed in GOM Inspect to calculate trueness and precision. Statistical evaluation included Shapiro-Wilk tests and paired t-tests ( = 0.05).

**Results:**

Only the Medit i700 scanner showed significantly improved trueness with geometrical landmarks for both cross-arch (P = 0.0011) and inter-cylindrical (P = 0.0060) measurements. Primescan AC and Trios 5 scanners showed no significant differences between GL and NG conditions (P &gt; 0.05). Visual analyses supported these findings, with Medit i700 benefiting from landmarks, while Primescan AC and Trios 5 maintained high trueness regardless of scanning strategy.

**Conclusions:**

The addition of geometrical landmarks improved scan trueness significantly for the Medit i700 scanner but had no measurable effect on Primescan AC or Trios 5. These findings suggest that the influence of auxiliary landmarks is scanner-dependent and should be considered when optimizing scanning protocols for edentulous arches.

## Introduction

Digital impressions using intraoral scanners (IOS) have become increasingly prevalent in prosthodontics, offering advantages such as improved patient comfort, time efficiency, and digital workflow integration. However, accurately capturing edentulous arches remains challenging due to the absence of distinct anatomical landmarks, which are essential for maintaining spatial orientation during scanning (1 - 3). This often results in cumulative errors that compromise the accuracy of full-arch impressions (4 , 5). This has been corroborated in recent literature, which highlight persistent challenges in capturing edentulous arches with intraoral scanners due to soft tissue mobility and the absence of fixed landmarks (6 , 7). Several in vitro studies have evaluated the trueness of intraoral scanners in edentulous scenarios. Braian and Wennerberg reported that IOS trueness varied significantly depending on whether the arch was dentate or edentulous, and also between scanner systems (8). Similarly, Schimmel et al. and Mangano et al. found that edentulous and implant-supported models are particularly sensitive to differences in scanner performance and scanning strategy, which may affect clinical fit (9 , 10). To address these limitations, the use of artificial geometrical landmarks has been proposed as a means of enhancing scan reliability by providing consistent reference geometry (11). While this approach has shown promise in improving image stitching and alignment, its efficacy may be scanner-dependent and remains under-explored in a standardized in vitro setting (12). Furthermore, recent reviews have highlighted the clinical potential of intraoral scanners in removable prosthodontics, noting variability in scanner performance across edentulous cases (13). This study focuses specifically on trueness, defined as the closeness of agreement between the mean of repeated measurements and a known reference value, in accordance with ISO 5725-1 (14). In vitro validation against a coordinate measuring machine (CMM) allows for precise evaluation of IOS performance under controlled conditions. Therefore, the aim of this in vitro study was to evaluate the trueness of three intraoral scanners and assess the impact of added geometrical landmarks on the digitization of edentulous mandibular complete-arch casts using a standardized scanning protocol.

## Material and Methods

**Figure 1:**
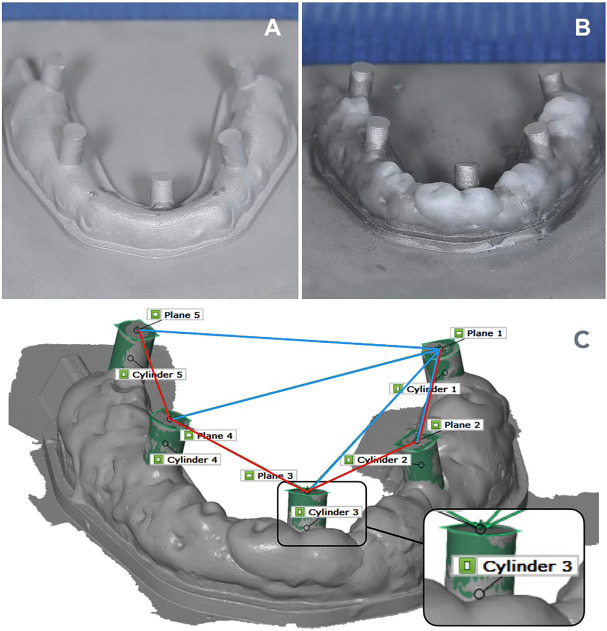
Mandibular cast used in the study. A) No additional geometry (NG), showing only cylindrical landmarks. B) Geometrical landmarks (GL) created by applying Visco-Gel between cylinders. C) 3D model illustrating cross-arch (blue) and inter-cylindrical (red) measurements, with inset showing the fitted cylinders, planes, and intersection points used for analysis.

Validation Cast Preparation A single edentulous mandibular cast was utilized for this study. Five cylindrical landmarks (height: 3 mm, diameter: 2 mm) were positioned at standardized locations to facilitate metrological measurements: Bilateral second molars (P1, P5) Bilateral second premolars (P2, P4) Lingual to the anterior teeth (P3) The cast was additively fabricated using a ConceptLaser M-lab 100W printer (GE Additive, Lichtenfels, Germany) with Remanium-Star-CL (Dentaurum GmbH &amp; Co. KG, Ispringen, Germany) alloy powder (Co 60.5 %, Cr 28 %, W 9 %, Si 1.5 %). Prior to printing, the cast was hollowed and optimized for errors using Materialise Magics v13 (Materialise NV, Leuven, Belgium). It was printed directly on a 90×90×7 mm build plate without support structures, with a layer height of 30 µm. Post-processing included heat treatment according to manufacturer guidelines and airborne particle abrasion with 250 µm aluminium oxide to achieve a non-reflective surface. - Geometrical Landmarks The model was scanned in two sessions. For the first session there was no additional geometries between the cylinders and for the second session Visco-Gel (Dentsply Sirona, Charlotte, NC, USA) was manually applied to the cast to form the geometrical landmarks by creating irregular surface geometries between each cylinder (Fig. 1).

1

**Figure 2:**
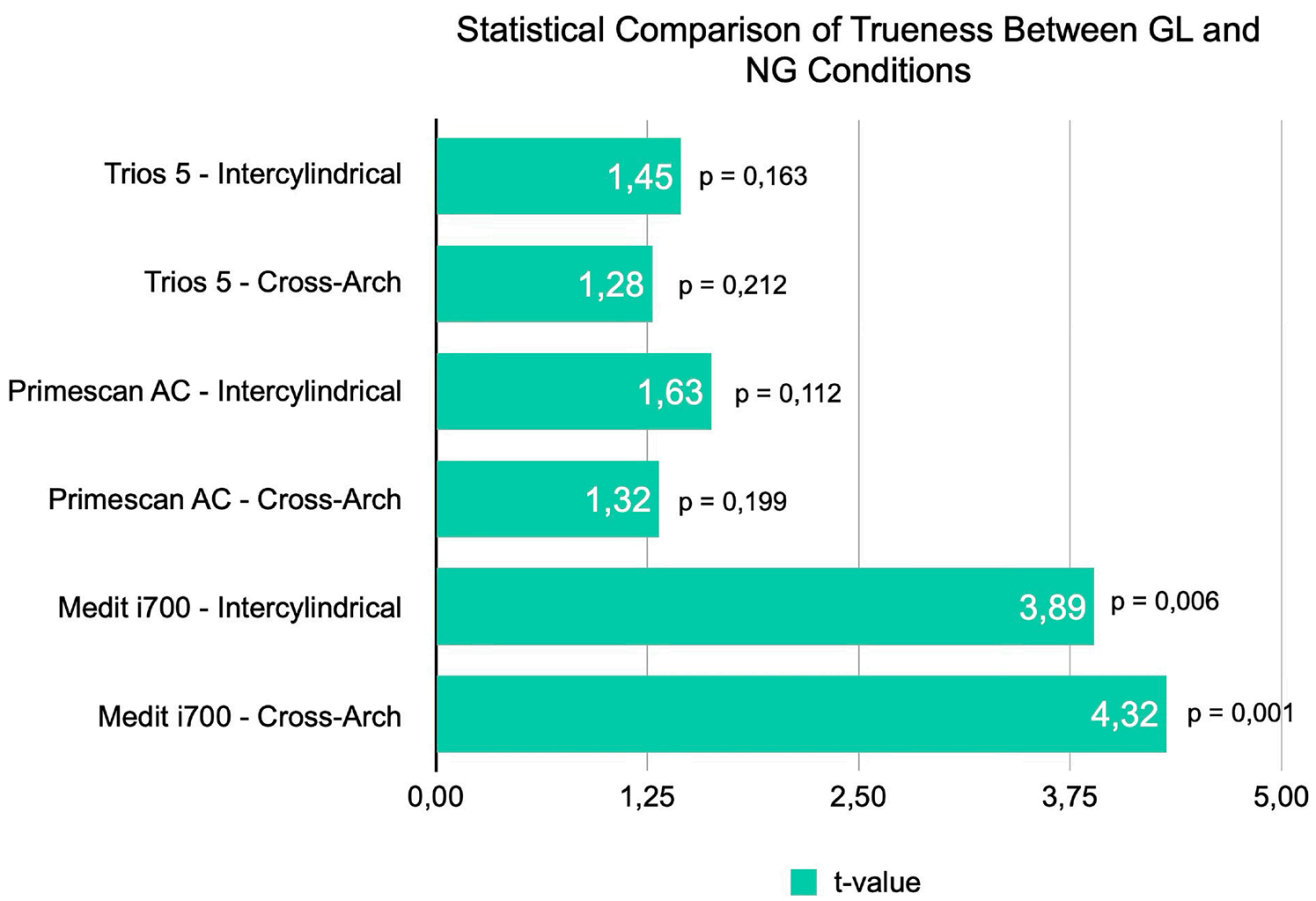
Statistical comparison of trueness for Medit i700, Primescan AC, and Trios 5 in cross-arch and inter-cylindrical measurements. P-values from paired t-tests indicate significance at α = 0.05.

This landmark approach is consistent with prior proposals for improving scan guidance in edentulous jaws (15 , 16). - Coordinate Measuring Machine (CMM) Validation The edentulous cast was measured using a Zeiss O-inspect CMM (153862; Carl Zeiss Industrielle Messtechnik GmbH, Oberkochen, Germany) at an ISO 13485:2016-certified facility (Elos Medtech). The CMM recorded the diameter and plane geometry of each cylinder and the intersection points between the cylinders and the corresponding planes. - Measurements included: Cross-arch distances: P1-P2, P1-P3, P1-P4, P1-P5 Inter-cylindrical distances: P1-P2, P2-P3, P3-P4, P4-P5 These CMM measurements served as the reference "true values" for subsequent comparisons. The reliability of this method for assessing IOS accuracy in edentulous applications has been validated in previous studies (17). - Intraoral Scanning Protocol Three intraoral scanners (IOS) were evaluated: Medit i700 (Medit Corp., Seoul, South Korea): MEDIT Link v3.3.3 Primescan AC ((Dentsply Sirona, Bensheim, Germany): Connect SW v5.2.8 Trios 5 (3Shape TRIOS A/S, Copenhagen, Denmark): 3Shape TRIOS v24.3.14/R1.0.3 A single calibrated operator performed all scans in a standardized environment (20 ± 1°C, 200 ANSI lumens). The cast was scanned 15 times per IOS under two conditions: without landmarks (NG) and with Visco-Gel landmarks (GL). The scanning sequence began at P1 and continued sequentially through P2, P3, P4, and P5. For cross-arch measurements, distances were calculated between P1 and P2, P1 and P3, P1 and P4, and P1 and P5. For inter-cylindrical measurements, distances were calculated between P1 and P2, P2 and P3, P3 and P4, and P4 and P5 (Fig. 2).

2

A 10-minute break was observed between scans to minimize potential thermal effects. All scans were exported as standard tessellation language (STL) files using the respective scanner software without third-party conversion. - Data Acquisition and Analysis STL files were imported into GOM Inspect 2017 (Carl Zeiss GOM Metrology GmbH, Braunschweig, Germany) for 3D deviation analysis against the CMM reference data. In GOM Inspect, the cylinders on the scanned mesh were converted into geometrical cylinders using GOM constructions; a similar approach was used to derive corresponding planes. The intersection points of these constructed geometries were then used to calculate the cross-arch and inter-cylindrical distances. Trueness was calculated as the mean absolute deviation (µm) between IOS measurements and the CMM reference values, and precision was determined by the standard deviation (SD) of the 15 repeated scans per condition (GL/NG). - Statistical Methods The Shapiro-Wilk test was used to assess data distribution for normality. Paired t-tests ( = 0.05) were conducted to evaluate differences between GL and NG conditions for each scanner and measurement type. All statistical analyses and data aggregation were performed using Microsoft Excel (Microsoft Corp., Redmond, WA, USA). - Ethics Statement All scans were performed on a single standardized edentulous mandibular cast under controlled laboratory conditions. No human or animal subjects were involved; therefore, ethics approval or waiver was not required for this study.

## Results

**Figure 3:**
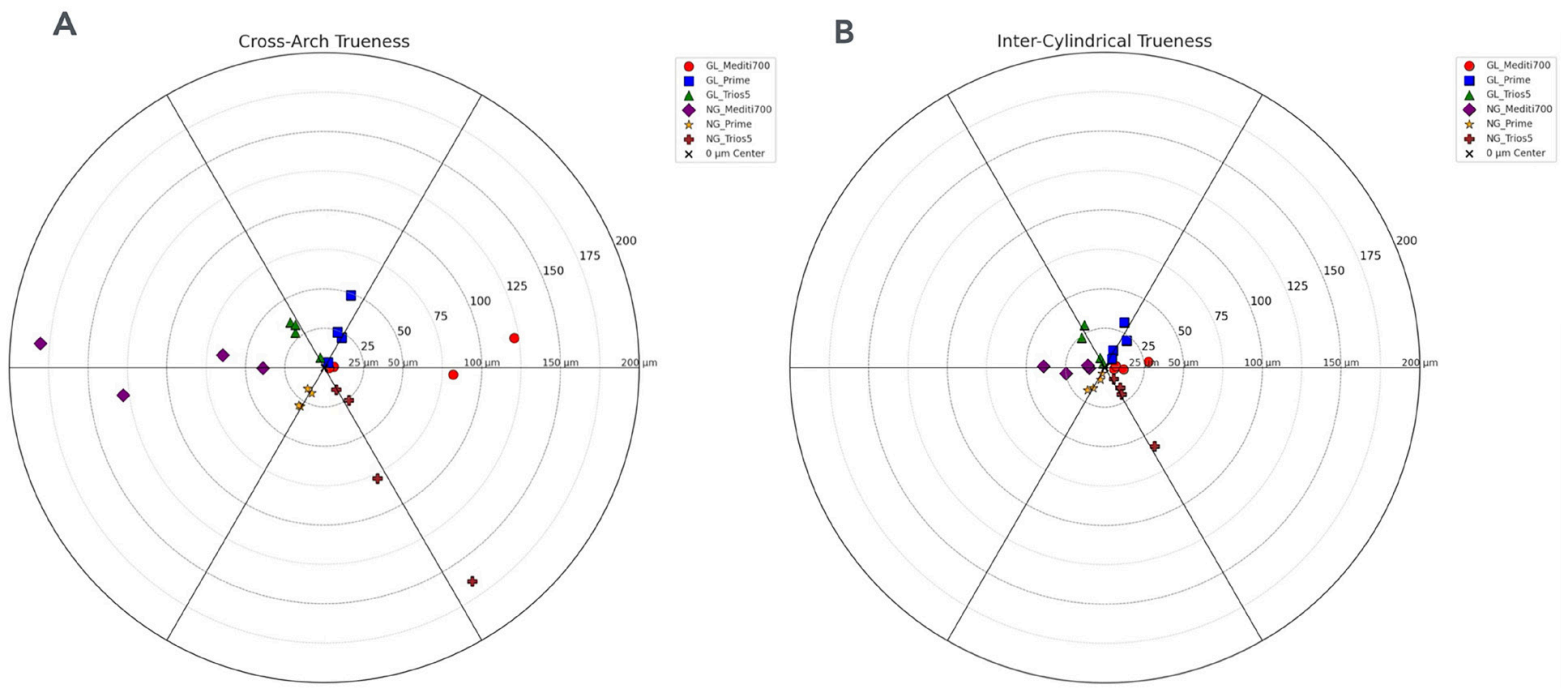
A) Bullseye chart showing cross-arch trueness across scanners and scanning protocols (GL/NG). B) Bullseye chart for inter-cylindrical trueness. Concentric rings (0–200 µm) and sector labels represent scanner and protocol combinations. Points closer to the center indicate higher trueness.

The trueness of intraoral scans obtained with geometrical landmarks (GL) was compared against scans without landmarks (NG) for three intraoral scanners (Medit i700, Primescan AC, and Trios 5) using two measurement types: cross-arch and inter-cylindrical distances. Data normality was confirmed using the Shapiro-Wilk test, and paired t-tests were then performed at a significance level of P &lt; 0.05. For the Medit i700 scanner, the paired t-tests revealed that the use of geometrical landmarks significantly improved trueness for both measurement types. Specifically, cross-arch measurements showed a significant difference (P = 0.0011), while inter-cylindrical measurements were also significantly improved (P = 0.0060). In contrast, for the Primescan AC, neither the cross-arch (P = 0.1988) nor inter-cylindrical (P = 0.1119) measurements demonstrated significant differences between GL and NG conditions. These findings align with recent studies indicating that some intraoral scanners are inherently more robust in maintaining scan fidelity regardless of scan strategy or surface modification (18). Similarly, for the Trios 5 system, cross-arch measurements did not differ significantly between the two scanning protocols (P = 0.2120). (See Figure 3 for a comprehensive statistical comparison across the scanners.), (Table 1).

3

Table 1

Figure 3 provide bullseye charts that graphically display deviations from the true values for cross-arch and inter-cylindrical measurements, respectively.

**Figure 4:**
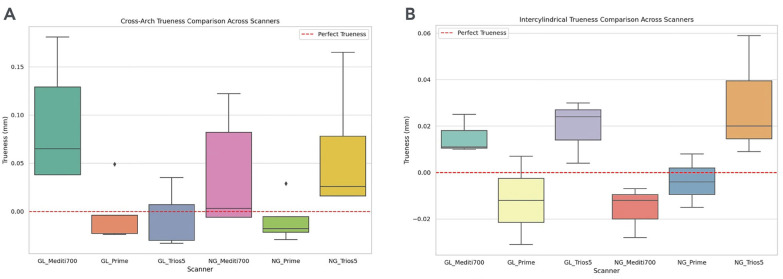
A) Trueness deviations (mm) for complete-arch scans grouped by scanner and scanning protocol (GL/NG). B) Inter-cylindrical trueness under the same conditions. Red dashed lines represent the ideal value (0 mm).

These figures are based on the detailed numerical data provided in the Measurements.xlsx file, which underpins the accuracy assessments of the scans. To further summarize the distribution of trueness data, box plots were constructed following best practices. Figure 4 presents box plots for cross-arch measurements across all scanner configurations (Medit GL, Medit NG, Primescan GL, Primescan NG, Trios 5 GL, and Trios 5 NG).

4

Each box plot displays the median deviation as a central line, the interquartile range (IQR) as the box limits, and the whiskers indicating the overall range of the data, with any outliers marked individually. Likewise, Figure 4 shows box plots for inter-cylindrical measurements. These visualizations highlight the central tendency and variability of the measurements and underscore the impact of geometrical landmarks on scan trueness.

## Discussion

The results showed that geometrical landmarks significantly improved scan trueness for the Medit i700 scanner, whereas no significant effect was observed for the Primescan AC and Trios 5 scanners. These findings are consistent with previous research indicating that IOS performance can vary considerably depending on scanner technology, software algorithms, and surface geometry (19). In edentulous arches, where anatomical features are less defined and surface texture is uniform, additional geometrical elements may assist in maintaining stitching accuracy during the scanning process. The improvement observed only for the Medit i700 suggests that certain IOS systems may be more responsive to surface guidance cues than others. Previous work by Mizumoto et al. has highlighted the potential of auxiliary landmark structures - such as resin or wax markers - to improve scan accuracy in edentulous arches by enhancing image stitching and spatial control (20). In a related clinical study, Al Hamad and Al-Kaff found that scan trueness was particularly problematic in the mandibular arch, likely due to soft tissue mobility and lack of vertical support, which may explain the scanner-dependent differences observed in the present study (21). Interestingly, the Primescan AC and Trios 5 scanners demonstrated high trueness regardless of the presence of geometrical landmarks. This suggests that certain intraoral scanners may inherently possess robust image acquisition and stitching algorithms that minimize reliance on surface features. From a clinical perspective, this could simplify the scanning procedure in edentulous cases by reducing the need for additional surface preparations, leading to a more streamlined and time-efficient workflow. This observation is in line with previous reviews that have reported consistent accuracy across scanning strategies for certain IOS platforms (22). While the study design ensured standardization and control through in vitro conditions and use of a coordinate measuring machine (CMM) as reference, it also introduces limitations. Intraoral conditions such as saliva, patient movement, and soft tissue compression were not replicated, which may affect clinical translatability. Limitations and future research While the study design ensured standardization and control through in vitro conditions and the use of a coordinate measuring machine (CMM) as reference, it also introduces inherent limitations. The use of a single standardized edentulous mandibular cast does not replicate intraoral conditions such as saliva, patient movement, soft tissue mobility, and border molding, all of which may affect scan accuracy and clinical translatability. Consequently, the external validity of the present findings is limited. Future research should include in vivo studies with edentulous patients to validate the observed scanner-dependent effects under clinical conditions and to explore optimized landmark designs and automated application methods. Despite these limitations, the study adds to the current understanding of how artificial landmarks affect scan accuracy. It also highlights the need for scanner-specific validation when modifying clinical scanning protocols. Future research should explore landmark optimization, automated application methods, and clinical in vivo validation of these findings. Additionally, recent reviews have proposed surface management strategies and marker-based techniques to improve scanning performance in edentulous arches, which may serve as a foundation for future innovation in scan protocol design (23). Recent narrative reviews have also outlined a variety of clinical and technical strategies - ranging from marker placement to scanning sequence adjustments - to improve the accuracy of digital impressions in completely edentulous arches (24).

## Conclusions

The use of geometrical landmarks significantly improved scan trueness for the Medit i700 scanner in both cross-arch and inter-cylindrical measurements. No significant effect was observed for the Primescan AC and Trios 5 scanners. The ability of the Primescan AC and Trios 5 scanners to maintain high trueness without the need for additional landmarks may contribute to a more efficient and less technique-sensitive scanning process in edentulous complete-arch cases. Tailoring scanning strategies to the specific characteristics of each intraoral scanner could help optimize clinical outcomes.

## Figures and Tables

**Table 1:** Table Mean trueness (µm), standard deviation (SD), effect size (Cohen’s d), and 95% confidence intervals (CI) for scans with (GL) and without (NG) geometrical landmarks.

Scanner	Condition	Mean trueness (µm)	SD (µm)	Cohen’s d(GL-NG)	95% CI
Primescan AC	NG	-8.0	18.6	-	-
	GL	-7.4	25.9	0.03	-0.70 to 0.76
Medit i700	NG	55.6	57.2	-	-
	GL	12.2	39.6	-0.90	-1.67 to -0.13
Trios 5	NG	48.1	64.5	-	-
	GL	6.1	47.3	-0.78	-1.54 to -0.02

SD: standard deviation; NG: no geometrical landmarks; GL: geometrical landmarks; µm: micrometers.

## Data Availability

The datasets used and/or analyzed during the current study are available from the corresponding author.
